# Improved Detection of Domoic Acid Using Covalently Immobilised Antibody Fragments

**DOI:** 10.3390/md11030881

**Published:** 2013-03-14

**Authors:** María J. Hortigüela, J. Gerard Wall

**Affiliations:** 1 Microbiology, NUI Galway, University Road, Galway, Ireland; E-Mail: mjhortiguela@gmail.com; 2 Network of Excellence for Functional Biomaterials (NFB), NUI Galway, University Road, Galway, Ireland

**Keywords:** domoic acid, scFv antibody fragment, covalent immobilisation, protein engineering, cysteine

## Abstract

Antibody molecules, and antibody fragments in particular, have enormous potential in the development of biosensors for marine monitoring. Conventional immobilisation approaches used in immunoassays typically yield unstable and mostly incorrectly oriented antibodies, however, resulting in reduced detection sensitivities for already low concentration analytes. The 2H12 anti-domoic acid scFv antibody fragment was engineered with cysteine-containing linkers of two different lengths, distal to the antigen binding pocket, for covalent and correctly oriented immobilisation of the scFvs on functionalised solid supports. The *Escherichia coli*-produced, cysteine-engineered scFvs dimerised in solution and demonstrated similar efficiencies of covalent immobilisation on maleimide-activated plates and minimal non-covalent attachment. The covalently attached scFvs exhibited negligible leaching from the support under acidic conditions that removed almost 50% of the adsorbed wildtype fragment, and IC_50_s for domoic acid of 270 and 297 ng/mL compared with 1126 and 1482 ng/mL, respectively, for their non-covalently adsorbed counterparts. The expression and immobilisation approach will facilitate the development of stable, reusable biosensors with increased stability and detection sensitivity for marine neurotoxins.

## 1. Introduction

Of the 5000 phytoplankton species known to date, approximately 300 can give rise to algal blooms and 40 species, which produce marine toxins, harmful algal blooms (HABs). In Europe, the estimated loss to the tourism and shellfish industries from algal blooms is estimated to be in the region of €900 million [1,2] while algal toxins, including amino acids, alkaloids and polyketides, are thought to be responsible for approximately 60,000 intoxications of humans worldwide each year [3].

Domoic acid (DA) is a water-soluble amino acid and the principal cause of amnesic shellfish poisoning (ASP) in humans. It is produced by diatoms of the genus *Pseudo-nitzschia* and accumulates mainly in the digestive glands of filter-feeding shellfish and fin fish such as anchovies and sardines that feed on the phytoplankton that produce the toxin (reviewed in [4]). Unlike marine toxins such as okadaic acid and azaspiracid that cause diarrheic shellfish poisoning and azaspiracid shellfish poisoning, respectively, and are associated only with gastrointestinal symptoms, ingestion of foods contaminated with DA can lead also to neurological complications [5,6]. Typical gastrointestinal symptoms of DA ingestion include nausea, cramps, vomiting and diarrhea, while in the case of neurological involvement, headaches, dizziness, ataxia and loss of memory can appear from a few hours to a few days after ingestion. In extreme instances, death can result [7].

In order to protect consumers and reduce the economic costs associated with algal toxins, regulatory authorities in the EU, USA and elsewhere have established relevant permitted levels [8]; in the case of DA, this is 20 mg DA/kg shellfish, though discussions are ongoing to lower this to 4.5 mg DA/kg shellfish [9,10]. The main platforms used to detect DA in shellfish samples are bioassays and biochemical or chemical approaches [11,12]. In the former group, the commonly used mouse toxicity assay raises obvious ethical concerns and is expensive, not sufficiently sensitive to meet regulatory needs [11] and subject to false positives and negatives [13]. A variety of quantitative chemical assays based on chromatographic techniques and mass spectrometry have been widely used for DA detection and measurement in laboratory environments (reviewed in [11]). The low limits of detection (down to pg/mL or ppb) and inter-assay reproducibility of such approaches is counterbalanced by the fact that they are time-consuming, relatively expensive and specialised to carry out, and not well suited to sample analysis in high-throughput or *in situ* settings. Antibody-based tests such as enzyme-linked immunosorbent assays (ELISA/EIA) offer a fast, simple-to-use, easily automated and inexpensive platform to detect and quantify DA in environmental samples with sensitivities that meet regulatory guidelines [12,14]. Immunobiosensors in particular provide immobilised antibodies (or, increasingly, inexpensively produced recombinant antibody fragments) that are suited to rapid marine monitoring *in situ*, including toxin tracking applications [15,16].

Effective immobilisation of the antibody moiety is critical in the development of robust immunosensing devices to detect low concentrate analytes. The antibody should be stably attached to the support to avoid leaching and facilitate sensor re-use, unmodified by the immobilisation strategy, form a monolayer on the sensor surface to avoid blocking of ligand-binding sites by other antibodies, and be correctly oriented to maximise accessibility of analyte-binding pockets [17]. We have previously reported the isolation and characterisation of a single-chain Fv (scFv) antibody fragment specific for DA [18] and its immobilisation on mesoporous silicate supports for use in DA detection [19,20]. In this study, we describe the design and development of an approach to covalently attach and improve orientation of the scFv on a functionalised solid support to improve the sensitivity and stability of DA detection. The strategy has broad potential application in biosensing of marine toxins.

## 2. Results and Discussion

### 2.1. Cloning and Sequencing of Cysteine-Functionalised scFvs

**Figure 1:**
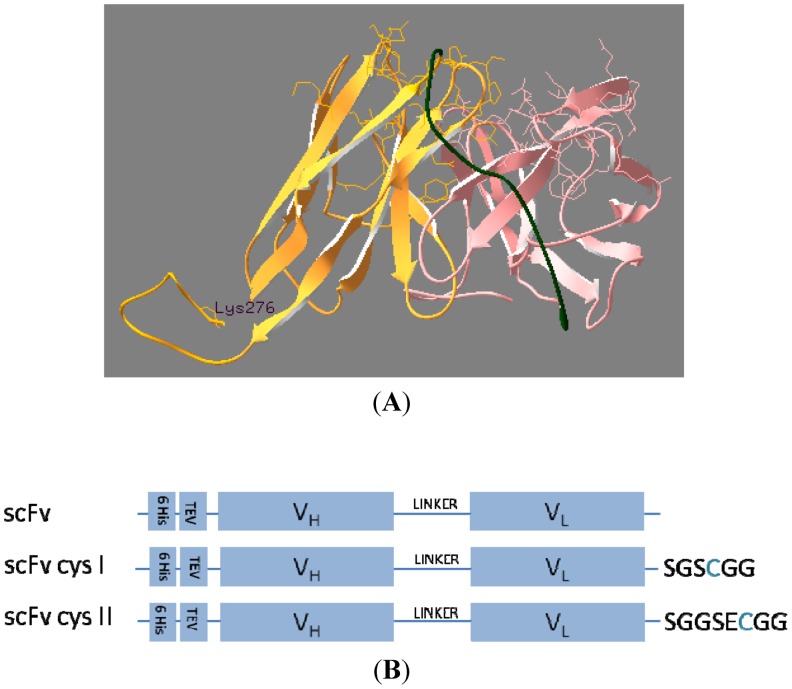
(**A**) Model of 2H12 scFv fragment. The V_H_ domain is coloured in pink, the V_L_ in orange and the inter-domain (Gly_4_Ser)_3_ peptide linker connecting the V_H_ and V_L_ domains in green. Side-chains of V_H_ and V_L_ amino acids that form part of the complementarity-determining regions (CDRs) [21] are shown and the antigen binding pocket is arrowed. The V_L_ *C*-terminal lysine residue 276 to which the cysteine-containing linkers were attached is labelled. (**B**) Schematic of the scFvs expressed in the study. scFv: single-chain Fv fragment containing the 2H12 V_H_ and V_L_ domains; scFv-cys I: scFv containing additional cysteine residue in a 6-amino acid *C*-terminal peptide linker; scFv-cys II: scFv containing additional cysteine residue in an 8-amino acid *C*-terminal peptide linker; 6His: hexahistidine tag; TEV: Tobacco Etch Virus (TEV) protease cleavage site; V_H_ and V_L_: antibody fragment heavy and light chain variable regions, respectively; linker: (Gly_4_Ser)_3_ polypeptide connecting V_H_ and V_L_ domains. The amino acid sequences of the engineered *C*-terminal linkers containing the additional cysteine residue (highlighted) are shown.

Homology-based prediction of the 2H12 scFv structure identified the scFv *C*-terminus as the most suitable location for placement of the cysteine-containing peptide tag in order to achieve correctly oriented immobilisation of the scFv without affecting conformation of, or access to, its antigen binding pocket (Figure 1A). Two constructs with flexible cysteine-containing tags of differing linker lengths at the *C*-terminal end were designed and constructed to investigate potential effects of the linker on antibody fragment dimerisation and/or steric requirements for attachment to the solid support. The scFv-encoding genes, containing an *N*-terminal *ompA* leader peptide for secretion of the translated polypeptide to the *Escherichia coli* periplasm, an adjacent hexahistidine tag for detection and purification of the scFv and the relevant cysteine-containing tag at the 3′-end, were generated and cloned into the pIG6 vector to express the proteins shown in Figure 1B, followed by confirmation of construct sequences prior to carrying out protein expression.

### 2.2. ScFv Expression and Purification

Optimisation of the previously described expression of the unmodified 2H12 scFv [19] using an autoinduction approach [22] revealed a 48-h expression period to yield the highest amounts of soluble 2H12 scFv protein.

**Figure 2:**
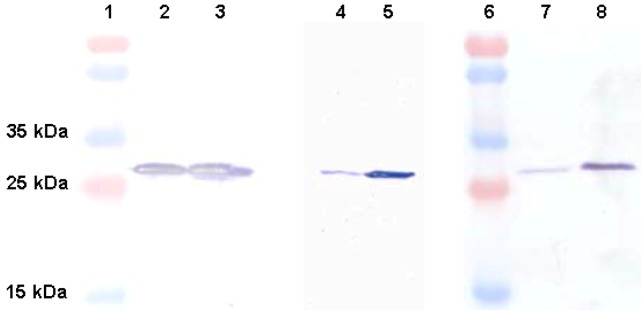
Comparison of expression of scFv variants in *E. coli*. Lanes 1,6: molecular weight markers; Lanes 2,3: soluble, insoluble 2H12 scFv produced under autoinduction conditions in *E. coli* BL21(DE3) for 48 h; Lanes 4,5: soluble, insoluble 2H12 scFv-cys I (autoinduction, 24 h, *E. coli* TOP10); Lanes 7,8: soluble, insoluble 2H12 scFv-cys II (autoinduction, 24 h, *E. coli* TOP10).

Expression of the 2H12 scFv-cys I and -cys II constructs was initially carried out using both IPTG and autoinduction approaches. While increasing amounts of the two scFvs accumulated throughout a 1–5 h IPTG induction period, greater than 95% of the protein molecules were found to be insoluble upon protein fractionation and immunoblot analysis, and no scFv was detectable upon purification (not shown). Under autoinducing conditions, a comparison of expression in *E. coli* TOP10 and *E. coli* BL21(DE3) strains revealed the highest soluble yields of both scFvs to be obtained after 24 h in *E. coli* TOP10 (Figure 2). While approximately 50% of all wildtype 2H12 scFv polypeptides produced in the *E. coli* periplasm were found to occur in a soluble, and therefore potentially active form, both scFvs with the added cysteine linker exhibited a large majority of insoluble polypeptides under all conditions investigated (Figure 2). This is most likely due to the occurrence of cross-linked, disulfide-bonded aggregates of scFv polypeptides, mediated by the additional cysteine residues, which remains unbridged in the native protein monomers. Work in our group with a similar scFv-cys I and -cys II construct pair based on a different scFv identified a significant improvement in the proportion of soluble scFv protein upon co-expression of a panel of Hsp60 and Hsp70 molecular chaperones, leading to increases in volumetric yields that exceeded that of the wildtype scFv by up to 10-fold. This work, as well as numerous demonstrations of the successful co-overexpression of *E. coli* disulfide bond isomerase C (DsbC) [23,24,25] or the eukaryotic protein disulfide isomerase (PDI) [26,27] to greatly improve expression or folding of multiple-disulfide-containing proteins in *E. coli* (reviewed in [28]), provides obvious opportunities to increase the active yields of the cysteine-containing scFv formats of interest in this work also. Further modification of expression parameters might also be beneficial as other researchers have reported four-fold higher yields of a cysteine-functionalised scFv under biofermenter compared with shake flask conditions, while, surprisingly, the yield of the unmodified scFv remained unchanged in the same comparison [29].

**Figure 3:**
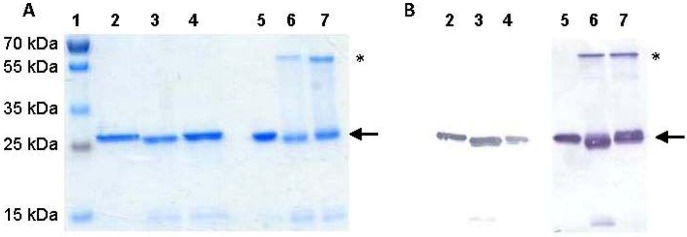
Investigation of disulfide-mediated dimerisation of 2H12 scFvs. (**A**) Coomassie blue-stained SDS-PAGE and (**B**) Immunoblot analysis of disulfide reduced (lanes 2–4) and non-reduced (lanes 5–7) purified 2H12 scFv (lanes 2,5), 2H12 scFv-cys I (3,6) and 2H12 scFv-cys II (4,7). Lane 1: Molecular weight marker. Samples in lanes 2,3,4 were loaded in reducing conditions; samples in lanes 5,6,7 were loaded in non-reducing conditions. Arrows indicate the monomeric scFv; asterisks indicate dimeric scFv molecules.

ScFvs were purified using immobilised metal affinity chromatography (IMAC), followed by an additional ion exchange step to remove smaller sized, apparent degradation products that appeared during purification of the 2H12 scFv-cys I and -cys II variants. While no evidence was found of significant degradation after purification of the scFvs, purified proteins were nonetheless frozen immediately after elution and used as soon as possible thereafter. This also reduced the potential for dimerisation of the scFvs via their unpaired cysteine residues prior to immobilisation analysis on maleimide-activated plates. Final yields of purified scFvs, adjudged by SDS-PAGE to be greater than 95% pure in all cases (Figure 3), were 3.15 mg/L, 294 μg/L and 337 μg/L for the unmodified, -cys I and -cys II scFvs, respectively. The approximately 10-fold lower soluble yields of the cys-containing mutants results from a combination of lower expression levels, degradation of the scFvs and the considerably greater proportion of the cysteine-containing proteins that is produced in the form of insoluble aggregates and is therefore inaccessible for purification. The accumulation of insoluble, aggregated scFv polypeptides, as well as their degradation, might be reduced by co-expression of molecular chaperones or folding catalysts as this approach has been demonstrated to improve the folding, and thus functional yield, of numerous poorly expressed heterologous proteins in *E. coli* (reviewed in [28])—including the 2H12 scFv [19]. As expression of multiply-disulfide-linked proteins [30,31] and of recombinant proteins with cysteine residues close to the *C*-terminus [32] are known to be problematic in bacteria, it is not unexpected that manipulation of chaperones *in vivo* might be required to achieve expression levels of the functionalised scFvs more comparable with those attainable with the wildtype scFv. Co-expression of disulfide-modifying enzymes in particular might prove productive in optimising expression of the cysteine-functionalised scFvs. 

In order to investigate the accessibility of the added cysteine residues in the folded scFvs, the abilities of the three scFvs to form dimeric molecules were analysed. While no dimers of the wildtype scFv were detectable, significant and similar levels of dimerisation were observed with the scFv-cys I and scFv-cys II proteins under non disulfide-reducing conditions (Figure 3). This indicated that both cysteine-containing linkers supplied the added cysteine residue in an accessible location in the native scFv conformation, suitable for inter-molecular interactions to form scFv dimers or with compatible chemical moieties on a solid support for oriented immobilisation of the scFvs.

### 2.3. ScFv Immobilisation and Domoic Acid Binding

**Figure 4:**
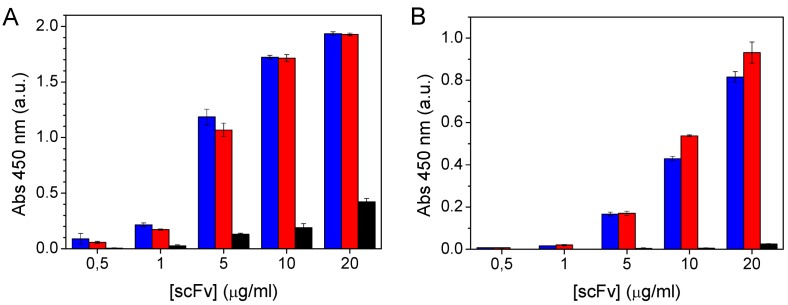
(**A**) Immobilisation of scFvs on maleimide-activated plates and (**B**) Domoic acid binding activity of immobilised scFvs. The immobilisation and binding of wildtype 2H12 scFv (black bars), 2H12 scFv-cys I (red) and 2H12 scFv-cys II (blue) is illustrated. Results shown are the average of triplicate samples and error bars represent the standard deviation.

The ability to immobilise the scFvs on a solid support via their cysteine-containing linkers and retain their ability to bind domoic acid was confirmed by EIA. Purified scFvs were dialysed in sodium phosphate buffer (pH 7.2) containing 150 mM NaCl and 10 mM EDTA prior to incubation in maleimide-activated plates as maleimide groups react with free sulfhydryls at pH 6.5–7.5 to form stable thioether linkages but reaction with amines becomes significant at higher pH values. Antibody fragments bound to the activated surface were detected using an anti-hexahistidine-HRP antibody conjugate, while DA binding of the immobilised scFvs was measured using a domoic acid-HRP tracer. The results demonstrated immobilisation of the cysteine-tagged antibodies on the activated surface but little or no attachment of the untagged scFv (Figure 4A). Upon normalisation for immobilised protein concentration, as determined by detection of the scFv using the anti-hexahistidine antibody, the scFv-cys I construct containing the shorter linker exhibited slightly higher DA binding than the scFv with the extended peptide linker at all protein concentrations tested (Figure 4B), though this may be due to differences in purity or the levels of degradation of the respective scFv preparations.

**Figure 5:**
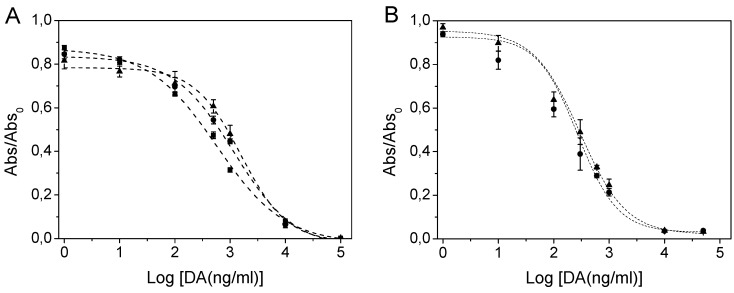
Comparison of domoic acid binding of (**A**) adsorbed and (**B**) covalently attached, oriented 2H12 scFvs. (**A**) ■ wildtype scFv on polystyrene; ● scFv-cys I on polystyrene; ▲ scFv-cys II on polystyrene. (**B**) ● scFv-cys I on maleimide-activated surface; ▲ scFv-cys II on maleimide-activated surface.

The domoic acid detection ability of the covalently immobilised antibody fragments was compared with that of scFvs adsorbed non-covalently onto polystyrene plates. The IC_50_ for DA of the unmodified 2H12 scFv in solution was previously reported as 245 ng/mL [18] whereas in this study, the 2H12 scFv, 2H12 scFv-cys I and 2H12 scFv-cys II proteins adsorbed onto standard Maxisorp (Nunc) plates were calculated to have IC_50_s for DA of 540 ng/mL, 1126 ng/mL and 1482 ng/mL, respectively. The lower sensitivity of the unmodified 2H12 scFv in its immobilised state is unsurprising as many proteins, including antibodies, typically lose some of their activity when immobilised, due to a combination of denaturation of the protein and steric hindrance of the binding site by multi-layering of proteins. In the case of antibodies, this can manifest itself as a reduction from a typical *K_d_* of the order of 10^−10^ to 10^−9^ mol/L in solution to in the region of 10^−7^ to 10^−5^ when immobilised. The slightly higher IC_50_ values determined for the cysteine-functionalised scFvs may be due in part to the higher level of protein degradation consistently observed in these proteins (Figure 3A), though a slightly increased IC_50_ has been reported by others for a similarly cysteine-modified scFv fragment [33]. For the cysteine-functionalised scFvs immobilised on the maleimide activated plates, however, the calculated IC_50_s were 270 ng/mL and 297 ng/mL for the scFv-cys I and scFv-cys II protein variants, respectively. These represent 4.2–5 fold improvements over their respective IC_50_s upon non-covalent adsorption and 1.8- to 2-fold improved over the adsorbed, unmodified scFv (Figure 5), indicating the impact of correct orientation on domoic acid binding and, potentially, detection sensitivity. Meanwhile the limit of quantification (LOQ), defined conservatively as the IC_20_ [34] was also determined for each scFv. In the case of non-covalently adsorbed scFvs, the LOQ was 80.1 ng/mL, 205.7 ng/mL and 412.6 ng/mL DA for the wild-type, scFv-cys I and scFv-cys II constructs, respectively, while for the covalently immobilised scFv-cys I and scFv-cys II, LOQs of 28.0 and 41.4 ng/mL, respectively, were determined for DA.

The stability of the immobilised scFvs was investigated under high salt and low pH conditions. Reductions in the domoic acid binding of non-covalently adsorbed scFvs were measured after a 1-h incubation at room temperature in phosphate buffer containing 1 M NaCl (DA binding reduced by 6.9%), or 100 mM acetate buffers of pH 5.0 (46.2%) or pH 3.6 (67%). While the high ionic strength used was expected to have a greater effect on protein desorption, the relatively minor effect of washing in high salt concentration may be due to the high concentration of salt (137 mM NaCl) present in the typical PBS-Tween wash buffer used to wash all plates prior to analysis. Adsorption of antibodies on a silica surface has been reported to be significantly reduced at ionic strengths of 100 and, particularly, 150 mM [35]. Meanwhile, pH is known to affect both dynamic adsorption and the equilibrated amount of adsorbed protein [35], while antibodies are capable of forming alternate stable structures, corresponding to neither the native folded form nor the denatured polypeptide, at pH values below 3 [36]. The reduction in antigen binding signal at pH 3.6 might, therefore, be the result of a combination of scFv desorption and denaturation.

**Figure 6:**
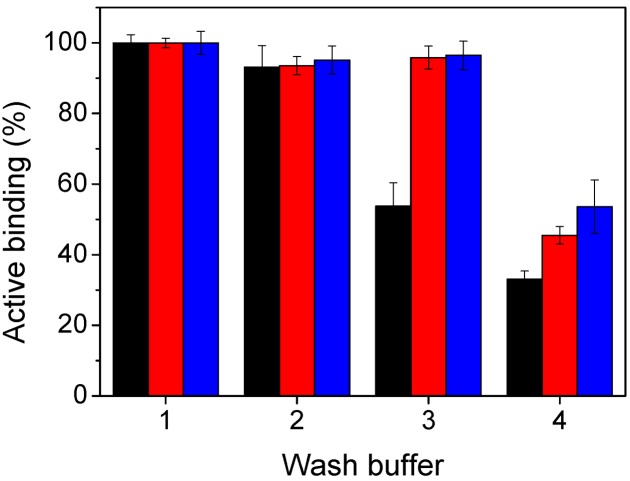
Stability of domoic acid binding by immobilised scFv fragments. Immobilised scFvs (black: wildtype 2H12 scFv; red: scFv-cys I; blue: scFv-cys II) were washed for 1 h at room temperature with: (1) Normal wash buffer (PBS containing 0.05% (v/v) Tween 20, pH 7.4); (2) 100 mM phosphate buffer containing 1 M NaCl, pH 7.2; (3) 100 mM acetate buffer, pH 5.0; (4) 100 mM acetate buffer, pH 3.6. Upon completion of the domoic acid-binding assay, DA binding was expressed as a percentage of the binding measured after washing with normal washing buffer. The wildtype scFv was immobilised on Maxisorp plates, the cysteine-modified scFvs on maleimide-functionalised plates.

The covalently attached cysteine-scFvs both exhibited greatly improved stability in the pH 5.0 acetate buffer, with reductions in DA binding of 6.4%, 4.1% and 54.5% for the scFv-cys I variant in 1 M NaCl, pH 5.0 and pH 3.6 buffers, respectively, and 4.8%, 3.5% and 46.4% for the scFv-cys II protein (Figure 6). While formation of the covalent disulfide bond between the scFv cysteine and the plate maleimide group is not favoured at low pH as the reaction equilibrium is shifted to favour the reduced, -SH form of cysteine, pre-formed disulfide linkages will be stable upon exposure to the low pH wash and so the desorption observed in the case of the unmodified fragment should not occur with the cysteine-functionalised scFvs. Therefore, the significant reduction in DA binding ability of the cysteine-modified scFvs in pH 3.6 buffer, which was comparable to that measured for the unmodified scFv, may be due to denaturation—and reduced antibody-antigen binding as low pH values, typically pH 2.5–3, effectively dissociates most antibody-antigen interactions and is a widely used approach to elute antibody-bound ligands in affinity chromatography. At pH 5.0, however, the stability of the cysteine-modified scFvs indicates the critical increase in stability of their covalent attachment to the plate walls. This stability of tethering of the antibody to the functionalised support has important potential in avoiding multi-layering of non-covalently attached proteins that reduce accessibility of the binding pocket and so leads to reduced sensitivity, as well as increased costs in sensor production. It may also facilitate the development of regenerable sensors in which the use of acidic pH could be exploited to elute toxins prior to re-use, or supports could be regenerated for use with antibodies of different binding specificities by immobilising fragments via cleavable, aldrithiol-type cross-linkers. Recent demonstrations of the functionalisation of diatom frustules containing very high surface areas with unbound, surface-exposed cysteines *in vivo* [37,38] also opens up enormous potential to further improve the sensitivity of detection of toxins through increasing the density of immobilised antibody fragments in stable biosensors suitable for *in vitro* or *in situ* monitoring.

## 3. Experimental Section

### 3.1. Materials, Strains and Plasmids

All chemicals were obtained from Sigma-Aldrich unless otherwise specified. Maleimide-activated 96-well plates, supplied pre-blocked with BSA, were from Pierce. *E. coli* TOP10 (F^−^ *mcr*A Δ(*mrr*^−^ *hsd*RMS^−^ *mcr*BC) φ80*lac*ZΔM15 Δ*lac*X74 *nup*G *rec*A1 *ara*D139 Δ(*ara*^−^*leu*)7697 *gal*E15 *gal*K16 *rps*L(Str^R^) *end*A1 λ^−^; Invitrogen) was used for cloning and genetic manipulation. ScFv expression was carried out in *E. coli* TOP10 or *E. coli* strain BL21(DE3) (F^−^ *omp*T *gal dcm lon hsd*S_B_(r_B_^−^ m_B_^−^) λ(DE3 [*lac*I *lac*UV5-T7 gene 1 *ind*1 *sam*7 *nin*5]); Invitrogen) using the pIG6 expression vector, a kind gift from Prof. Andreas Plückthun, University of Zurich, Switzerland.

### 3.2. Genetic Construction

Cysteine-encoding linkers were added to the 3′ end of the 2H12 scFv gene in the pIG6 vector [19] by incorporation into oligonucleotides for PCR amplification of the antibody fragment gene. Oligonucleotides used were 2H12_SGSCGG: ggtc*aagctt*tcagccaccgcaagaaccactccgttttatttc (*HindIII* site for cloning in italics; added *C*-terminal SGSCGG underlined) and 2H12_SGGSECGG: ggtc*aagctt*tcagccaccgcattcagacccaccgctccgttttatttc (*HindIII* site in italics; added *C*-terminal SGGSECGG underlined). This generated scFvs in the expression format *ompA* leader-His_6_-TEV cleavage site-2H12 scFv-cys tag upon cloning into the pIG6 plasmid (Figure 1). Gene sequences were confirmed prior to expression of the scFv variants under the control of the plasmid-encoded *lac* promoter in *E. coli*.

### 3.3. ScFv Modelling

A homology-based prediction of the 2H12 scFv fragment structure was generated using the iterative threading assembly refinement (I-TASSER) server [39,40]. The co-ordinates of the top model were imported into DeepView Swiss-Pdb Viewer [41] for image analysis. CDRs were identified based on the Kabat numbering system [21].

### 3.4. ScFv Expression and Purification

For autoinduction experiments, *E. coli* clones containing the relevant plasmid were grown at 37 °C in 5 mL of LB media containing 100 μg/mL ampicillin until an OD_600_ of 0.9–1 was reached. After inoculation of the starter culture into 500 mL of ZYM-5052 autoinducing medium [22] containing 100 μg/mL ampicillin, cultures were grown, with shaking, at 25 °C for 48 h (2H12 scFv) or 24 h (2H12 scFv-cys I and 2H12 scFv-cys II).

For preparation of periplasmic proteins, cells were harvested (8000 rpm, 15 min) and pellets washed with 500 mL of cold PBS and resuspended in 15 mL of ice-cold buffer containing 750 mM sucrose, 100 mM Tris-HCl, pH 7.5. Following drop-wise addition of 30 mL of ice-cold 1 mM EDTA, the mixture was incubated at room temperature for 10 min. rLysozyme^®^ (30 U) was added and the solution incubated for a further 30 min at room temperature, followed by 1 h on ice before the addition of 4.3 mL of 250 mM MgCl_2_. The viscosity of the reaction mixture was reduced by the addition of 50 μg/mL of DNaseI for 15 min on ice and the solution was centrifuged at 12,000 rpm for 15 min. After dialysis of the supernatant against 5 L purification buffer (20 mM phosphate, 500 mM NaCl, pH 7.5) at 4 °C overnight, it was filtered through a 0.45-μm filter prior to the addition of Tween 20 to 2% (v/v) and imidazole to 10 mM. The sample was applied at a flow rate of 1 mL/min to a 1-mL Hitrap prepacked column (Amersham Biosciences, UK) that had been charged with Ni^2+^ ions and pre-equilibrated with the same buffer. The column was washed with purification buffer containing 20, 40 and 60 mM imidazole to remove non-specifically adsorbed materials and proteins were eluted in 1-mL buffer fractions containing 500 mM imidazole. After buffer exchange using a desalting column (P 10, GE Healthcare), cysteine-tagged scFvs were immediately applied to a 1-mL Resource™ Q ion exchange column (GE Healthcare) in 20 mM ethanolamine (pH 9.5) for further purification and eluted in the same buffer containing increasing concentrations of NaCl. Purified proteins were dialysed against 2 L of PBS buffer, or 100 mM phosphate, 150 mM NaCl, 10 mM EDTA buffer (pH 7.2) in the case of proteins to be immobilised on maleimide-activated plates. Purified proteins were analysed by SDS-PAGE, Coomassie staining and Western blot detection using an anti-polyhistidine horse radish peroxidase (HRP)-conjugated antibody [19].

### 3.5. Binding Studies

#### 3.5.1. Immobilisation of scFvs on Maleimide-Activated Plates

Purified, dialysed scFvs (100 μL aliquots of 20, 10, 5, 1 or 0.5 μg/mL protein samples) were added to the wells of a maleimide-activated plate and incubated overnight at 4 °C with gentle shaking. After three washes with 200 μL buffer containing 100 mM phosphate, 150 mM NaCl, 0.05% (v/v) Tween 20 (pH 7.2), wells were blocked with 200 μL of 10 μg/mL cysteine in the same buffer for 1 h at room temperature. Wells were washed three times, followed by the addition of 100 μL anti-polyhistidine HRP-conjugated antibody (1:3000 in PBS) or domoic acid-HRP tracer (1:20 in supplied tracer dilution buffer; Mercury Science) for 1 h at room temperature with gentle shaking. After three washes, 100 μL of 3,3′,5,5′-tetramethylbenzidine (TMB) solution was added to each well and reactions were stopped by the addition of 100 μL of 1 M H_2_SO_4_. Absorbances were read at 450 nm.

#### 3.5.2. Competitive EIA in Polystyrene Plates

The wells of a 96-well microtitre plate (Nunc Maxisorp) were coated overnight at 4 °C with 50 μL of the relevant 2H12 scFv solution at 10 μg/mL in 100 mM carbonate-bicarbonate buffer (pH 9.6). After three washes with PBS containing 0.05% Tween 20, pH 7.4 (PBST), wells were blocked for 2 h at room temperature using 200 μL of 2% BSA in PBS. Following three washes with PBST, 50 μL of domoic acid solution at concentrations from 1 to 100,000 ng/mL in PBS was added to each well and plates were agitated gently for 1 h at room temperature. Domoic acid-HRP tracer (50 μL, 1:10 dilution in supplied tracer dilution buffer) was added to each well and plates were agitated gently at room temperature for 1 h prior to five washes with PBST. TMB (50 μL) was added to develop the reaction, which was stopped using 50 μL of 1 M H_2_SO_4_. Absorbances were read at 450 nm. OriginPro8 was used to fit the data according to the theory of competitive binding and to calculate the deduced IC_50_ values for the scFvs.

#### 3.5.3. Competitive EIA in Maleimide-Activated Plates

The wells of a maleimide-activated plate were coated overnight at 4 °C with 100 μL of 2H12 scFv-cys I or 2H12 scFv-cys II (15 μg/mL in binding buffer containing 100 mM phosphate, 150 mM NaCl, 10 mM EDTA, pH 7.2). After three washes with buffer containing 100 mM phosphate, 150 mM NaCl, 0.05% (v/v) Tween 20 (pH 7.2), wells were blocked using 10 μg/mL cysteine in the same binding buffer for 1 h at room temperature and washed three times as before. Domoic acid solutions (100 μL) ranging from 1 to 50,000 ng/mL were incubated for 1 h at room temperature with gentle shaking and 50 μL of the reaction mixture was replaced in each well by 50 μL of domoic acid-HRP tracer solution. After incubation for 1 h at room temperature with shaking, wells were washed three times as before and 100 μL TMB was added. Reactions were stopped by the addition of 100 μL of 1 M H_2_SO_4_ and absorbances were read at 450 nm after transfer of the reaction solution to a non-activated microtitre plate. IC_50_ values were calculated for scFvs as described above.

### 3.6. Immobilisation Studies

For maleimide-activated plates, the relevant cys-tagged 2H12 scFv (20 μg/mL in 100 mM phosphate, 150 mM NaCl, 10 mM EDTA buffer, pH 7.2) was incubated in plate wells overnight at 4 °C with gentle shaking. This was followed by washing of wells for 1 h at room temperature with one of four wash regimes: “wash buffer” containing 100 mM phosphate, 150 mM NaCl, 0.05% (v/v) Tween 20 (pH 7.2); 100 mM phosphate buffer containing 1 M NaCl (pH 7.2); 100 mM acetate buffer (pH 5); or 100 mM acetate buffer (pH 3.6). Wells were then blocked with 200 μL of 10 μg/mL cysteine for 1 h at room temperature followed by three washes in wash buffer and incubation with 100 μL of domoic acid-HRP tracer (1:20 dilution) for 1 h at room temperature. After three further washes, the reaction was developed using 100 μL of TMB solution, stopped with 100 μL of 1 M H_2_SO_4_, and absorbances were read at 450 nm after transfer of the reaction solution to a non-maleimide-activated microtitre plate.

For non-covalent adsorption studies with the unmodified 2H12 scFv, the wells of a 96-well microtitre plate (Nunc Maxisorp) were coated overnight at 4 °C with gentle shaking with 50 μL of the purified scFv (10 μg/mL). Wells were washed for 1 h at room temperature with one of the four buffers outlined above and blocked with 200 μL of 10 μg/mL cysteine for 1 h at room temperature. After three washes in wash buffer, 50 μL domoic acid-HRP tracer (1:20 dilution) was added prior to incubation for 1 h at room temperature. Three washes were followed by the addition of 50 μL of TMB and stopping of the reaction using 50 μL of 1 M H_2_SO_4_. Absorbances were read at 450 nm.

## 4. Conclusions

The 2H12 anti-domoic acid scFv antibody fragment was engineered with cysteine-containing linkers of two different lengths, distal from the antigen binding pocket. The cysteine-modified scFvs exhibited similar efficiencies of covalent, oriented immobilisation on maleimide-activated plates and minimal non-covalent attachment. The covalently immobilised scFvs had IC_50_ values for domoic acid similar to the unmodified 2H12 in solution, overcoming the effects on antigen binding of uncontrolled orientation and protein-surface interactions in the case of adsorbed scFvs. The scFvs also demonstrated improved stability at acidic pH when covalently attached compared with absorbed molecules, with higher levels of domoic acid binding retained after incubation with acidic buffers. This antibody functionalisation and immobilisation approach will facilitate the development of stable, reusable biosensors for *in vitro* or *in situ* analysis of marine neurotoxins.

## References

[1] Morgan K.L., Larkin S.L., Adams C.M. (2009). Firm-Level economic effects of HABS: A tool for business loss assessment. Harmful Algae.

[2] Hoagland P., Scatasta S., Graneli E.T.T. (2006). The Economic Effect of Harmful Algal Blooms. Ecology on Harmful Algae.

[3] Van Dolah F.M., Ramsdell J.S. (2001). Review and assessment of *in vitro* detection methods for algal toxins. J. AOAC Int..

[4] Mos L. (2001). Domoic acid: A fascinating marine toxin. Environ. Toxicol. Pharmacol..

[5] Debonnel G., Beauchesne L., de Montigny C. (1989). Domoic acid, the alleged “mussel toxin,” might produce its neurotoxic effect through kainate receptor activation: An electrophysiological study in the dorsal hippocampus. Can. J. Physiol. Pharmacol..

[6] Grant K.S., Burbacher T.M., Faustman E.M., Gratttan L. (2010). Domoic acid: Neurobehavioral consequences of exposure to a prevalent marine biotoxin. Neurotoxicol. Teratol..

[7] Quilliam M.A., Wright J.L. (1989). The amnesic shellfish poisoning mystery. Anal. Chem..

[8] Campbell K., Vilariño N., Botana L.M., Elliott C.T. (2011). A European perspective on progress in moving away from the mouse bioassay for marine-toxin analysis. TrAC Trends Anal. Chem..

[9] Gerssen A., Pol-Hofstad I.E., Poelman M., Mulder P.P., van den Top H.J., de Boer J. (2010). Marine toxins: Chemistry, toxicity, occurrence and detection, with special reference to the Dutch situation. Toxins.

[10] EFSA Panel on Contaminants in the Food Chain (2009). Scientific Opinion of the Panel on Contaminants in the Food Chain on a request from the European Commission on marine biotoxins in shellfish–domoic acid. EFSA J..

[11] He Y., Fekete A., Chen G., Harir M., Zhang L., Tong P., Schmitt-Kopplin P. (2010). Analytical approaches for an important shellfish poisoning agent: Domoic Acid. J. Agric. Food Chem..

[12] Vilariño N., Louzao M.C., Vieytes M., Botana L. (2010). Biological methods for marine toxin detection. Anal. Bioanal. Chem..

[13] Holland P., Botana L.M. (2008). Analysis of Marine Toxins—Techniques, Method Validation, Calibration Standards and Screening Methods. Seafood and Freshwater Toxins: Pharmacology, Physiology and Detection.

[14] Traynor I.M., Plumpton L., Fodey T.L., Higgins C., Elliott C.T. (2006). Immunobiosensor detection of domoic acid as a screening test in bivalve molluscs: Comparison with liquid chromatography-based analysis. J. AOAC Int..

[15] MacKenzie L.A. (2010). *In situ* passive solid-phase adsorption of micro-algal biotoxins as a monitoring tool. Curr. Opin. Biotechnol..

[16] Doucette G.J., Mikulski C.M., Jones K.L., King K.L., Greenfield D.I., Marin Iii R., Jensen S., Roman B., Elliott C.T., Scholin C.A. (2009). Remote, subsurface detection of the algal toxin domoic acid onboard the Environmental Sample Processor: Assay development and field trials. Harmful Algae.

[17] Hu X., O’Connor I.B., Wall J.G. (2012). Antibody Immobilization on Solid Surfaces: Methods and Applications. Biological Interactions with Surface Charge in Biomaterials.

[18] Hu X., O’Dwyer R., Wall J.G. (2005). Cloning, expression and characterisation of a single-chain Fv antibody fragment against domoic acid in *Escherichia coli*. J. Biotechnol..

[19] Hu X., O’Hara L., White S., Magner E., Kane M., Wall J.G. (2007). Optimisation of production of a domoic acid-binding scFv antibody fragment in *Escherichia coli* using molecular chaperones and functional immobilisation on a mesoporous silicate support. Protein Expr. Purif..

[20] Hu X., Spada S., White S., Hudson S., Magner E., Wall J.G. (2006). Adsorption and activity of a domoic acid binding antibody fragment on mesoporous silicates. J. Phys. Chem. B.

[21] Kabat E.A. (1983). Sequences of Proteins of Immunological Interest.

[22] Studier F.W. (2005). Protein production by auto-induction in high-density shaking cultures. Protein Expr. Purif..

[23] Bessette P.H., Qiu J., Bardwell J.C., Swartz J.R., Georgiou G. (2001). Effect of sequences of the active-site dipeptides of DsbA and DsbC on *in vivo* folding of multidisulfide proteins in *Escherichia coli*. J. Bacteriol..

[24] Zhang S.T., Shi J., Zhao J., Qi Y.F., Guo A.G. (2006). Expression of soluble and functional snake venom fibrinolytic enzyme fibrolase via the co-expression of DsbC in *Escherichia coli*. Protein Peptide Lett..

[25] Maskos K., Huber-Wunderlich M., Glockshuber R. (2003). DsbA and DsbC-catalyzed oxidative folding of proteins with complex disulfide bridge patterns *in vitro* and *in vivo*. J. Mol. Biol..

[26] Subedi G.P., Satoh T., Hanashima S., Ikeda A., Nakada H., Sato R., Mizuno M., Yuasa N., Fujita-Yamaguchi Y., Yamaguchi Y. (2012). Overproduction of anti-Tn antibody MLS128 single-chain Fv fragment in *Escherichia coli* cytoplasm using a novel pCold-PDI vector. Protein Expr. Purif..

[27] Liu Y., Zhao T.-J., Yan Y.-B., Zhou H.-M. (2005). Increase of soluble expression in *Escherichia coli* cytoplasm by a protein disulfide isomerase gene fusion system. Protein Expr. Purif..

[28] Kolaj O., Spada S., Robin S., Wall J.G. (2009). Use of folding modulators to improve heterologous protein production in *Escherichia coli*. Microb. Cell Fact..

[29] Albrecht H., Burke P.A., Natarajan A., Xiong C.-Y., Kalicinsky M., DeNardo G.L., DeNardo S.J. (2003). Production of soluble ScFvs with *C*-terminal-free thiol for site-specific conjugation or stable dimeric ScFvs on demand. Bioconjug. Chem..

[30] O’Dwyer R., Razzaque R., Hu X., Hollingshead S.K., Wall J.G. (2009). Engineering of cysteine residues leads to improved production of a human dipeptidase enzyme in *E. coli*. Appl. Biochem. Biotechnol..

[31] Kiedzierska A., Czepczynska H., Smietana K., Otlewski J. (2008). Expression, purification and crystallization of cysteine-rich human protein muskelin in *Escherichia coli*. Protein Expr. Purif..

[32] Ackerson C.J., Jadzinsky P.D., Jensen G.J., Kornberg R.D. (2006). Rigid, specific, and discrete gold nanoparticle/antibody conjugates. J. Am. Chem. Soc..

[33] Wang D., Berven E., Li Q., Uckun F., Kersey J.H. (1997). Optimization of Conditions for formation and analysis of Anti-CD19 FVS191 single-chain Fv homodimer (scFv’)2. Bioconjug. Chem..

[34] Shaw I., O’Reilly A., Charleton M., Kane M. (2008). Development of a high-affinity anti-domoic acid sheep scFv and its use in detection of the toxin in shellfish. Anal. Chem..

[35] Zhao X., Pan F., Garcia-Gancedo L., Flewitt A.J., Ashley G.M., Luo J., Lu J.R. (2012). Interfacial recognition of human prostate-specific antigen by immobilized monoclonal antibody: Effects of solution conditions and surface chemistry. J. R. Soc. Interface.

[36] Buchner J., Rudolph R., Lilie H. (2002). Intradomain disulfide bonds impede formation of the alternatively folded state of antibody chains. J. Mol. Biol..

[37] Yang Y., Addai-Mensah J., Losic D. (2012). Key factors influencing the optical detection of biomolecules by their evaporative assembly on diatom frustules. J. Mater. Sci..

[38] Fowler C.E., Buchber C., Lebeau B., Patarin J., Delacôte C., Walcarius A. (2007). An aqueous route to organically functionalized silica diatom skeletons. Appl. Surf. Sci..

[39] Roy A., Kucukural A., Zhang Y. (2010). I-TASSER: A unified platform for automated protein structure and function prediction. Nat. Protoc..

[40] Zhang Y. (2008). I-TASSER server for protein 3D structure prediction. BMC Bioinforma..

[41] Guex N., Peitsch M.C. (1997). SWISS-MODEL and the Swiss-PdbViewer: An environment for comparative protein modeling. Electrophoresis.

